# Simultaneous idiopathic segmental infarction of the great omentum and acute appendicitis: a rare association

**DOI:** 10.1186/1749-7922-3-30

**Published:** 2008-10-29

**Authors:** Luigi Battaglia, Filiberto Belli, Alberto Vannelli, Giuliano Bonfanti, Gianfrancesco Gallino, Elia Poiasina, Mario Rampa, Marco Vitellaro, Ermanno Leo

**Affiliations:** 1 Colo-rectal Cancer Surgery Unit, Department of Surgery, Fondazione IRCCS "Istituto Nazionale dei Tumori", via Venezian, 1 - Milan, 20133, Italy

## Abstract

Idiopathic segmental infarction of the greater omentum is an uncommon cause of acute abdomen. The etiology is still unclear and the symptoms mimic acute appendicitis. Its presentation simultaneously with acute appendicitis is still more infrequent. We present a case of a 47-year old woman without significant previous medical history, admitted with an acute abdomen, in which the clinical diagnosis was acute appendicitis and in whom an infarcted segment of right side of the greater omentum was also found at laparotomy. As the etiology is unknown, we highlighted some of the possible theories, and emphasize the importance of omental infarction even in the presence of acute appendicitis as a coincident intraperitoneal pathological condition.

## Review

Omental Infarction, the result of impaired perfusion to the greater omentum, is a rare entity. First described by Bush in 1896 [1], the incidence of idiopathic segmental infarction of the greater omentum is estimated to be 0.1% of the total laparotomies performed for acute abdomen [2].

Even though more than 100 cases have been reported in the literature, its association with acute appendicitis has been rarely documented. More interestingly, this case also presented in a female patient, which makes the observation far more uncommon.

The purpose of this report is to describe our first experience with this condition. Even though the etiology is unknown, we highlight some of the possible theories. Analysis of some collective reviews [3] and our experience with this patient indicate that the symptoms, clinical findings, preoperative diagnosis and management of this condition are almost identical, even in children.

A 47-year-old woman presented to the emergency department complaining of right lower abdominal pain of 48 hours of duration along with high fever and nausea without vomiting. The patient had no relevant previous medical history. The pain started at the right paraumbilical and subcostal region, radiating down to the right iliac fossa region.

Physical examination revealed normal vital signs, no fever, no abdominal distension but a tender abdomen in the right lower quadrant with guarding and rebound tenderness. No mass was palpable. Results of laboratory studies revealed leucocytocis with a WBC count of 12560/mm^3 ^and a CRP of 173 mg/L. Both, the plain x-rays and the ultrasonography of the abdomen showed no abnormalities. No further tests were performed, and the patient was taken to the operating room with the diagnosis of acute appendicitis after appropriate antibiotic prophylaxis was administrated.

**Figure 1:**
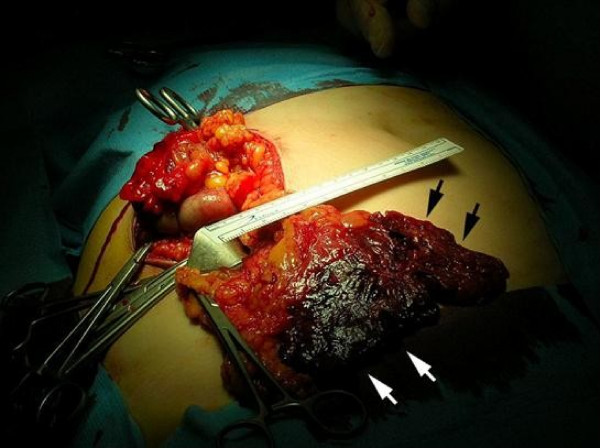
**Macroscopical appearance of the infarcted area of the greater omentum found during laparotomy for acute appendicitis.** Note the change in color and edema of the omental fat (arrows).

Surgery was performed through a right pararectal incision in order to allow a good examination of the of the ceco-appendicular and surrounding area. At surgery, the omentum was found to be grossly dark and thickened. Adhesions between the greater omentum and the right abdominal wall were seen. Further exploration revealed infarction without torsion of the right segment of the greater omentum localized at the inferior right quadrant region in an area of approximately 120 cm^2 ^(10 cm per 12 cm) (Fig. 1). The appendix was edematous and erythematous macroscopically, located in a preileal position. A small amount of serosanguineous fluid was seen at the right iliac fossa region.

The infarcted segment of the omentum was released from the surrounding adhesions, resected and removed. Further inspection showed no other abnormalities. Therefore, a partial omental resection and a base-apical appendectomy were performed.

**Figure 2:**
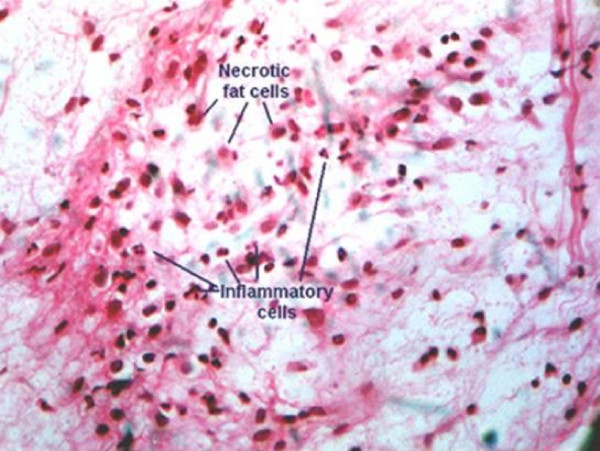
**Micrograph showing the histological results of the infarcted omentum.** Note the areas of fat necrosis and liquefactive changes. There are also scattered acute inflammatory cells.

Histopathological examination confirmed the diagnosis of omental infarction and phlegmonous acute appendicitis. The histological examination revealed a reddish infarction of the fatty tissue of the greater omentum. The omentum contained scattered hemorrhages and the vessels were markedly distended with blood. (Fig. 2)

The postoperative course was uneventful and the patient was discharged on the fifth postoperative day.

Omental infarction is an infrequent entity. Its presentation together with intraoperatively and histopathologically confirmed acute appendicitis is far more uncommon. To the best of our knowledge, this is the first case in the literature reporting such unusual association.

Depending on the causative factor, this clinical problem can be classified as omental infarction with torsion, and omental infarction without torsion, commonly known as idiopathic omental infarction (Table 1).

**Table 1:** Classification of the infarctions of the greater omentum.

***Omental infarction without torsion:***
Primary (Idiopathic infarction of the greater omentum)
Secondary: hernia, hypercoagulabily, pathology vascular, polyglobulia
***Omental infarction with torsion:***
Primary
Secondary: adherences, cysts, tumor

In a recent collective review of nineteen cases of the idiopathic type from the last 20 years [3], 42% of the cases occurred in pediatric patients. Female patients were affected only in 15% of the cases. Only 21% of the patients (4 out of 19) presented with fever, and the mean white blood cells count was 12.633/mm^3^. Almost 75% of the cases (14 out of 19) went to the operating room with a diagnosis of acute appendicitis, and 36% of all cases were resolved by laparoscopy. In one half of the cases, free serosanguineous peritoneal fluid was also present at exploration.

Among the published cases, our patient has a more atypical presentation that those previously reported. As described earlier, most authors suggest acute appendicitis as preoperative diagnosis, but the appendix is usually found to be macroscopically normal either at exploration or at histopathological analysis [4].

The exact etiology and pathogenesis of this condition is unknown. Some authors have suggested that congenitally anomalous fragile blood supply to the right lower portion of the greater omentum renders this region prone to infarction [5]. Other authors [6] suggest a different embryonic origin for the right side of the greater omentum with more fragile blood vessels which are more susceptible to elongation and secondary occlusions. Such a theory could explain the high incidence (90%) of this disease in the right side of the greater omentum. Variations in blood supply to the right omental edge associated with obesity or trauma, overeating, hypercoagulability, coughing or a sudden change in position have been suggested as predisposing factors [5]. Other authors suggested venous engorgement after heavy meals or venous elongation produced by excessive weight of the greater omentum as a cause, since the higher prevalence of the syndrome in the obese population [6].

Clinically, most patients present with acute or subacute abdominal pain. The pain may be to the left or right side of the midline based on the side of omental involvement. Pain may localize to the upper or lower quadrant of the abdomen, simulating acute appendicitis (66%) or cholecystitis [5]. In female patients, the entity can also mimic gynecologic problems.

The physical findings are variable but usually there is tenderness in the right side of the abdomen, predominantly at the right lower quadrant. Physical examination usually elicits localized tenderness with or without a palpable "mass". Temperature is usually normal or slightly raised. Occasionally, the WBC count may be elevated. Therefore, clinically, omental infarction is difficult to be distinguished from appendicitis, cholecystitis, or adnexal problems.

Since it is rarely diagnosed before surgery, the imaging features of omental resection have been seldom described in the radiological literature. Computed tomography and/or ultrasound can be extremely helpful in establishing the diagnosis. Both may show a well circumscribed, ovoid or cake-like soft tissue mass. Because of omental fat, the lesion is hyperechoic at ultrasound and of mixed attenuation due to fatty and no fatty elements on computed tomography [7].

Pathologically, the right side of the omentum is affected in most of patients. The histological appearance of omental infarction differs with the duration of insult. Initially, hemorrhagic infarction with fat necrosis is seen, followed by infiltration by lymphocytes, histiocytes, and finally, fibroblasts, resulting in fibrosis and scar formation.

Differentiation between torsion and infarction is not of practical significance as the management remains the same, i.e. surgical resection of the infarcted omentum, and it is the usual treatment when the diagnosis is not established preoperatively. Either by open surgery or laparoscopy, the rationale for excision rests on the theoretical possibility of adhesions forming about the infarct, which could obstruct nearby bowel loops.

Idiopathic segmental infarction of the right sided greater omentum should be considered even in the presence of acute appendicitis or other intra abdominal pathologies since it may occur and mimic the basic pathologic condition as an associated disease. Furthermore, even when other viscera are found to be normal at exploration, the omentum should be inspected for infarction, especially if free serosanguineous peritoneal fluid is present.

## Conclusion

It is possible that infarction of unknown origin involving the greater omentum is more common than is usually thought. We emphasize that idiopathic segmental infarction of the greater omentum should be included in the differential diagnosis of any patient with right sided abdominal pain, and inspection of the omentum should be a routine part of exploration when a more common cause of abdominal complaint is not readily obvious at operation or even in the presence of other intra abdominal conditions.

## Consent

"Written informed consent was obtained from the patient for the publication of this article and accompanying images. A copy of the written consent is available for review by the Editor-in-Chief of this journal."  

## Competing interests

The authors declare that they have no competing interests.

## Authors' contributions

LB wrote the manuscript and participated to surgical procedures and to preoperative and postoperative patient management, AV participated in manuscript design and coordination, GB participated in literature reviewing and patient follow-up, GG participated in literature reviewing and patient follow-up, EP participated in literature reviewing, MR participated in literature reviewing, MV participated in literature reviewing, FB participated in literature reviewing, EL is the Chief of the Surgical Unit and participated in manuscript design.
